# Editorial Comment: Comparative Analysis of Super-Mini Percutaneous Nephrolithotomy and Retrograde Intrarenal Surgery for the Management of Renal Calculi ≤2 cm Among Somali Population

**DOI:** 10.1590/S1677-5538.IBJU.2024.9918

**Published:** 2024-07-25

**Authors:** Luciano A. Favorito

**Affiliations:** 1 Universidade do Estado do Rio de Janeiro Unidade de Pesquisa Urogenital Rio de Janeiro RJ Brasil Unidade de Pesquisa Urogenital - Universidade do Estado do Rio de Janeiro - Uerj, Rio de Janeiro, RJ, Brasil; 2 Hospital Federal da Lagoa Rio de Janeiro RJ Brasil Serviço de Urologia, Hospital Federal da Lagoa, Rio de Janeiro, RJ, Brasil

**Abdihamid Hassan Hilowle** ^1^, **Abdikarim Hussein Mohamed** ^2^

**J Endourol. 2024 May;38(5):426-431.**

DOI: 10.1089/end.2023.0675 | ACCESS: 38299931

## COMMENT

The management of the urinary stones constitutes one of the most usual treatments in in urology practice. There are several options for the renal stones treatment: shockwave lithotripsy (SWL), retrograde intrarenal surgery (RIRS) and percutaneous nephrolithotomy (PCNL) ( 1 – 3 ). In the past, PCNL was done for large-volume stones such as complex multiple calyceal calculi, staghorn stones. The reduction of the tract size and the advent of miniaturization of instruments ushered in the development of smaller scopes, smaller retrieval devices, and energy sources were responsible for paradigm shift in the indications for PCNL ( 3 , 4 ). These miniaturized instruments and accessories obviated the need to dilate the tract beyond 20 Fr.

The newer techniques with Miniperc are suited for stones 1.5–2 cm in size. Microperc and Ultraminiperc may be suitable for stone sizes <1.5 cm ( 1 – 4 ). The present paper aims to comprehensively evaluate the safety and effectiveness of super-mini percutaneous nephrolithotomy (SMP) compared with RIRS ( 5 ). The authors studied 210 patients with renal calculi (≤2 cm) undergoing SMP or RIRS, randomly recruited over 4 years and concluded that SMP demonstrated superior efficacy with significantly shorter operating times and reduced hospital stays, suggesting potential advantages for managing lower volume renal stones.
